# Screening for Potential Active Components of Fangji Huangqi Tang on the Treatment of Nephrotic Syndrome by Using Integrated Metabolomics Based on “Correlations Between Chemical and Metabolic Profiles”

**DOI:** 10.3389/fphar.2019.01261

**Published:** 2019-10-22

**Authors:** Xiao Liu, Qi-Gang Zhou, Xiao-Chai Zhu, Li Xie, Bao-Chang Cai

**Affiliations:** ^1^School of Pharmacy, Nanjing University of Chinese Medicine, Nanjing, China; ^2^School of Pharmacy, Nanjing Medical University, Nanjing, China

**Keywords:** Fangji Huangqi Tang, metabonomics, correlation analysis, nephrotic syndrome, marker

## Abstract

As for traditional Chinese medicine (TCM) prescription, what puzzled researchers most was how to select proper chemical markers to represent the whole pharmacological action system. In this paper, an integrated metabolomic method was presented for a systematic discovery of potential active components in Fangji Huangqi Tang (FHT), a well-known TCM prescription for nephrotic syndrome treatment, based on “correlations between chemical and metabolic profiles.” Firstly, a metabolomics study was carried out to select representative biomarkers of nephrotic syndrome. Then, after drug administration, the dynamic process of serum composition was investigated by the ultra-high performance liquid chromatography coupled with electrospray ionization–quadrupole–time of flight–mass spectrometry (UHPLC-ESI-Q-TOF-MS) technique to detect the prototypes and related metabolites of relative components from FHT. Pearson correlation analysis was finally used to find out the correlations between the endogenous metabolic spectrums and the chemical serum spectrums. As a result, 17 biomarkers for nephrotic syndrome indication were identified, and the main metabolic pathways of their concern included linoleic acid metabolism; cyanoamino acid metabolism; alpha-linolenic acid metabolism; glycine, serine, and threonine metabolism; arachidonic acid metabolism; and glycerophospholipid metabolism. Meanwhile, active components in FHT for nephrotic syndrome treatment were screened out, including (+)-tetrandrine demethylation, fenfangjine G hydrogenation, tetrandrine, *N*-methylfangchinoline, tetrandrine demethylation, fangchinoline, glycyrrhetic acid, astragaloside II alcohol dehydration, atractylenolide III demethylation + hydrogenation, atractylenolide III demethylation + hydrogenation, and licoricone-*N*-acetylcysteine conjugation. This study demonstrated a promising way to elucidate the active chemical material basis of TCM prescription.

## Introduction

Nephrotic syndrome is a common disease of the urinary system, which could be divided into primary and secondary types, with clinical manifestations such as proteinuria, hypoalbuminemia, edema, and hyperlipidemia. Due to its complicated pathogeny and various pathological types, treatment of nephrotic syndrome is very difficult in the clinic (Crawford and Gipson, 2017; Kang et al., 2017). Compared with modern drugs, traditional Chinese medicine (TCM) had some special advantages in treating nephrotic syndrome, especially in the aspects of reducing proteinuria and side effects of immunosuppressive drugs, as well as alleviating the complications of hypercoagulability or gastrointestinal mucosa edema (Wang and Wang, 2004; Nie, 2008; Wu et al., 2014). Furthermore, compared with hormone, TCM application to treatment of nephrotic syndrome is also a good way to avoid side effects and to reduce high recurrence rate of disease.

The most characteristic clinical manifestation of nephrotic syndrome is edema. According to the theory of Chinese medicine, the reason for edema could be concluded to Qi Xu, especially caused by deficient spleen or kidney functions. In other words, the disorder of the spleen or kidney is an important factor during nephrotic syndrome development. Fangji Huangqi Tang (FHT), a classic TCM prescription in Chinese clinical application for the treatment of chronic glomerulonephritis, is composed of the roots of four kinds of Chinese herbal medicines, namely, *Stephania tetrandra* S. Moore (FJ), *Astragalus membranaceus* Fisch. ex Bunge (HQ), *Atractylodes macrocephala* Koidz. (BZ), and *Glycyrrhiza uralensis* Fisch. ex DC. (GC). A large number of literatures indicated that FHT showed a significant effect on nephrotic syndrome (Chen et al., 2013; Zhang and Yu, 2015; Liu et al., 2019). However, underlying active components and modern therapeutic mechanisms of FHT for nephrotic syndrome are still unclear.

The curative and protective effects of TCM have already been widely and fully recognized through clinical practice for thousands of years in China. However, the detailed bioactive components and potential mechanisms for most of TCM remained poorly understood. People know that using a single index or a simple superposition way to explain the multi-herb synergistic characteristics of TCM would not be sensible. In recent years, papers on metabolomics of TCM began to appear (Zhou et al., 2016; Cui et al., 2017; Pang et al., 2018), including report on TCM metabonomics for nephrotic syndrome treatment, by which the protective effect of total flavonoids in *Astragali Radix* against adriamycin-induced nephropathy was proved using rats (Zhang et al., 2018). Metabolomics technology and biomarkers were used in these papers to screen out active or toxic substances in TCM. However, this kind of work was not too much by now, and how to assess the efficacy of herbal drugs in a metabolomic way quantitatively is to some extent a challenging problem. In this paper, a novel strategy based on metabonomics combined with spectrum–effect research was proposed for FHT active chemicals investigation. The core academic thought of metabonomics (Jové et al., 2014; Patel and Ahmed, 2014; Bjerrum et al., 2015) was integrated into a classical spectrum–effect relationship research method. The variational biological levels of biomarkers *in vivo* were regarded as the indicators to reflect the pathological and physiological changes of the organism under the actions of drugs, by which way the traditional spectrum–effect relationship was translated to the relationships between drug chemical spectrums and serum metabolic profiles. Actually, this action of translation would benefit the researchers with a better reproducibility of the experimental data obtained from chemical spectrum analyses, as well as a lower financial cost because of the absence of biochemical index assay. It was hoped that this newly developed method would clarify the most active chemicals in FHT to treat nephrotic syndrome. Also, the elucidation of FHT acting material bases would be an innovative way for modern research of TCM formulas.

## Materials and Methods

### Chemicals and Reagents

Four component herbs of FHT were obtained as follows: *A. membranaceus* Fisch. ex Bunge (HQ) and *G. uralensis* Fisch. ex DC. (GC) were obtained from the Nanjing Haichang Chinese Medicine Group Corporation (Nanjing, China), and *S. tetrandra* S. Moore (FJ) and *A. macrocephala* Koidz. (BZ) were obtained from the Hebei Chinese Medicine Group Corporation (Hebei, China). These species were authenticated by Prof. Jianwei Chen (School of Pharmacy, Nanjing University of Chinese Medicine) for their botanical origin before use (**Supplementary Figures 1**–**4**). Voucher specimens (no. NZY-LIU-2019001 for FJ; no. NZY-LIU-2019002 for HQ; no. NZY-LIU-2019003 for BZ; and no. NZY-LIU-2019004 for GC) were deposited in the Herbarium of Traditional Chinese Medicine, School of Pharmacy, Nanjing University of Chinese Medicine, Nanjing, China. Acetonitrile and water were of LC-MS grade from Merck Company (Darmstadt, Germany). HPLC-grade methanol was purchased from ANPEL Scientific Instrument Co., Ltd. (Shanghai, China), as well as HPLC-grade formic acid with a purity of 99% (Anaqua Chemicals Supply, USA). All the other reagents were of analytical grade and obtained from the Nanjing Chemical Reagent Company (Nanjing, China).

### Decoction Preparation

According to the original composition and preparation process recorded in “Jin-Gui-Yao-Lue,” FHT was obtained following these procedures: dry herb pieces of *S. tetrandra* S. Moore (20 g), *A. membranaceus* Fisch. ex Bunge (25 g), *A. macrocephala* Koidz. (15 g), and *G. uralensis* Fisch. ex DC.(10 g) were mixed together in a certain ratio (4:5:3:2, *w*/*w*/*w*/*w*) and macerated in deionized water for 30 min before being decocted twice with boiling water (1:10, *w*/*v*, and 1:8, *w*/*v*, respectively), 20 min for each time. Then the solution was filtered through a two-layer mesh, combined and concentrated to a density of 1 g/ml for intragastric administration (*i.g*.).

The composition of FHT used in this paper was characterized by UHPLC-ESI-Q-TOF-MS chromatograms (Wang et al., 2015). Fangchinoline, atractylenolide I, tetrandrine, atractylenolide III, calycosin-7-glucoside, liquiritigenin, isoliquiritigenin, liquiritin, isoliquiritin, and glycyrrhizin were tested as the dominating compounds for obvious representational significance during this work for a description of the composition of FHT. The typical total ion chromatogram (TIC) and extract ion chromatogram for 10 major compounds of FHT in positive ion mode were shown in **Supplementary Figure 5**, and the chemical structures of the 10 selected major compounds of FHT were given out in **Supplementary Figure 6**.

### Animals and Drug Administration

Male Sprague-Dawley (SD) rats (*n* = 18), weighing 200–220 g, were supplied by the Slaccas Experiment Animal Company (Shanghai, China). Temperature and humidity conditions of the breeding environment were kept constant, with food and water provided *ad libitum*. All the rats were acclimated in the laboratory for at least 1 week prior to the experiment. Before testing, animals were fasted overnight with free access to water. All the animal experiments were carried out according to the Guidelines for the Care and Use of Laboratory Animals and were approved by the Animal Ethics Committee of Nanjing University of Chinese Medicine [approval number: ACU-13(20151120)].

Rats were randomly divided into a normal group (Group A, *n* = 6) and a model group (Group B, *n* = 12). A nephrotic syndrome model was prepared by giving rats injections of adriamycin (doxorubicin, 4 mg/kg) *via* the tail vein twice on the 1st and 14th days, respectively (Liu et al., 2019). The normal-group rats were injected with the same volume of saline instead. On the 0th (before injection) and 17th days after the first adriamycin injection, urine samples were collected from rats.

On the 22nd day, after the first adriamycin injection, all the rats in Group B were further randomly divided into an untreated model group (*n* = 6) and a test group (*n* = 6). The rats in the test group were administered with FHT at a dose of 14 g/kg (calculating by crude herb) for 5 weeks; meanwhile, the rats in the normal and model groups were given saline instead. Both FHT and saline were given to rats by gavage only once in 1 day. Blood samples (no more than 0.5 ml) of all these three groups of rats were collected from the orbital vein into tubes without being heparinized on 5th, 12th, 19th, 22nd, 23rd, 24th, 25th, 36th, 44th, 49th, 54th, and 57th days. The whole experiment period was 57 successive days.

Urinary protein excretion was determined by benzethonium chloride turbidimetric method using the urine samples within a period of 24 h in metabolic cages. Total cholesterol (TC), total triglyceride (TG), blood urea nitrogen (BUN), and cystatin C (Cys C) levels were measured by using commercially available kits based on colorimetric assays (Jian Cheng Biological Technology Co., LTD, Nanjing, China).

### Sample Preparation

Blood samples were kept for 30 min under room temperature and then centrifuged to get a good separation at 4,000 rpm for 5 min. The supernatant was collected and stored at −80°C until analysis.

Spiked into centrifuge tubes was 0.1 ml of serum, which was and then mixed with 500 μl of methanol by vortex mixing for 30 s. The mixture was separated by centrifugation at 12,000 rpm for 5 min. The upper layer was then transferred to another tube and evaporated to dryness under nitrogen at 40°C. The residue was reconstituted with 100 μl of methanol, vortexed for 30 s, and centrifuged at 12,000 rpm for 3 min. Finally, 2 μl of the supernatant was used for UHPLC-ESI-Q-TOF-MS analysis.

Meanwhile, 15 plasma samples were randomly selected from real serum samples and were mixed together as quality control (QC) samples. These QC samples contained the most data of each group. They were analyzed to equilibrate the UHPLC-ESI-Q-TOF-MS system and to monitor the stability of this method. All these samples were maintained at 4°C during analysis.

### UHPLC-ESI-Q-TOF-MS Conditions

For analysis of serum samples, an ekspert^™^ ultra LC 100-XL system coupled to hybrid quadrupole time-of-flight tandem mass spectrometry (LCMS-Triple TOF^™^ 5600, AB SCIEX, Foster City, CA) with an electrospray ionization (ESI) interface was used. The Analyst 1.6 software was installed for highly efficient data recording and processing.

Chromatographic separation was performed on a C_18_ reversed-phase UHPLC column (2.1 mm × 100 mm, 1.8 μm, Agilent). The column temperature was set at 30°C. The flow rate was 0.4 ml/min. The mobile phase consisted of (A) 0.1% formic acid in water and (B) acetonitrile. In order to obtain endogenous components with different polarities, the optimized gradient elution of metabonomics was set as follows: 0–15 min, 5–95% B; 15–16 min, 95% B; and 16–17 min, 95–5% B. At the same time, based on a previous study (Wang et al., 2015), the optimized gradient elution for FHT relative components detection in rat serum was as follows: 0–3 min, 17% B; 3–10 min, 17–70% B; 10–12 min, 70% B; 12–18 min, 70–80% B; 18–20 min, 80% B; 20–23 min, 80–100% B; 23–26 min, 100% B; 26–28 min, 100–17% B; and 28–30 min, 17% B.

The mass spectrometer was operated in positive ion modes. The following parameter settings were used: turbo spray temperature (TEM) of 550°C; declustering potential (DP) of 60 V; collision energy (CE) of 35 V; nebulizer gas (Gas 1) of 55 psi; heater gas (Gas 2) of 55 psi; and curtain gas of 35. Nitrogen was used as the nebulizer and auxiliary gas. TOF-MS and TOF-MS/MS were performed with the mass ranges of *m*/*z* 100–1,200 and 50–1,000, respectively. The experiments were running with a 200 ms accumulation time for TOF-MS and 80 ms accumulation time for TOF-MS/MS. Continuous recalibration was carried out every 3 h. In addition, dynamic background subtraction (DBS) and information-dependent acquisition (IDA) were applied to trigger acquisition of MS/MS for these constituents with extremely low signal levels.

### Biomarker Screening and Identification

Raw data collected by the UHPLC-ESI-Q-TOF-MS system were imputed into the MarkerView^™^ software for principal component analysis (PCA). Before that, the alignment and normalization of the chromatogram peaks were performed. The time range was 1–17 min, the scan range of quality was 100–1,000 *m*/*z*, the tolerance range of the mass number was 0.05, and the peak strength threshold was 100. The intensity of extracted ion was standardized by the total peak area method (normalized using total area sums). The samples collected on the 22nd day from the A and B groups were tested for screening differentiated endogenous metabolites. The identification of these selected biomarkers was accomplished by comparing the information from qualitative analysis and secondary mass spectrums and then confirmed by literatures as well as public databases including the Human Metabolome Database (HMDB), METLIN, and Small Molecular Pathway Database (SMPD). Furthermore, the identified metabolites were determined by a comparison with reference standards available in the lab. Also, the intensities of these selected biomarkers were detected and recorded.

### Dynamic Detection of Serum Composition

Post-acquisition analyses were performed using the PeakView^™^ 1.2 software, which employed an extensive list of information of fragmentation in combination with the elemental compositions of the substrate molecules to generate a series of extracted ion chromatograms (XICs). The accurate mass and composition information for the precursor ions and fragment ions were quickly analyzed. Furthermore, the analyses of metabolites and prototype components were performed using the MetabolitePilot^™^ 1.5 programs containing a variety of functions such as predicted metabolites, generic peak finding, isotope pattern, and mass defect, based on a previous work (Wang et al., 2015).

### Correlation Analysis Between Chemical and Metabolic Profiles

The basic idea of canonical correlation analysis (CCA) is to study the relationships between two sets of variations, as well as to determine the related degree. In this work, CCA was applied to find out the relationships between chemical and metabolic profiles. For each time point, the peak areas of relative components and their metabolites from FHT and the candidate biomarkers detected from rat serum samples were comprehensively calculated by using professional SPSS software for statistics (SPSS for Windows 17.0, SPSS Inc., USA).

## Results

### Results of Plasma Metabonomics

#### Nephrotic Syndrome Modeling

The biomedical indexes of rats for nephrotic syndrome indication in the normal and model groups, such as urinary protein, TC, TG, BUN, and Cys C, were investigated firstly to determine whether the nephrotic syndrome model was constructed successfully. The results are shown in **Supplementary Table 1**. Compared with those in the normal group, 24 h urine protein, TC, TG, BUN, and Cys C levels in the model group were obviously elevated, which indicated that the nephrotic syndrome model was successfully induced.

**Figure 1:**
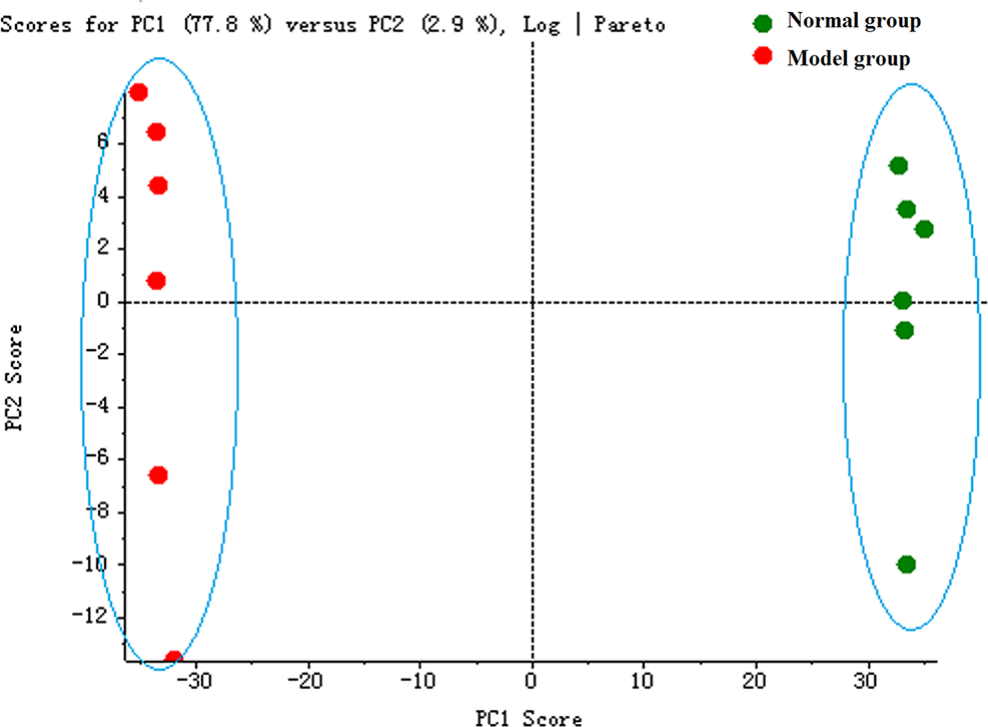
The principal component analysis (PCA) score plot of rat serum samples between normal and model groups.

**Figure 2:**
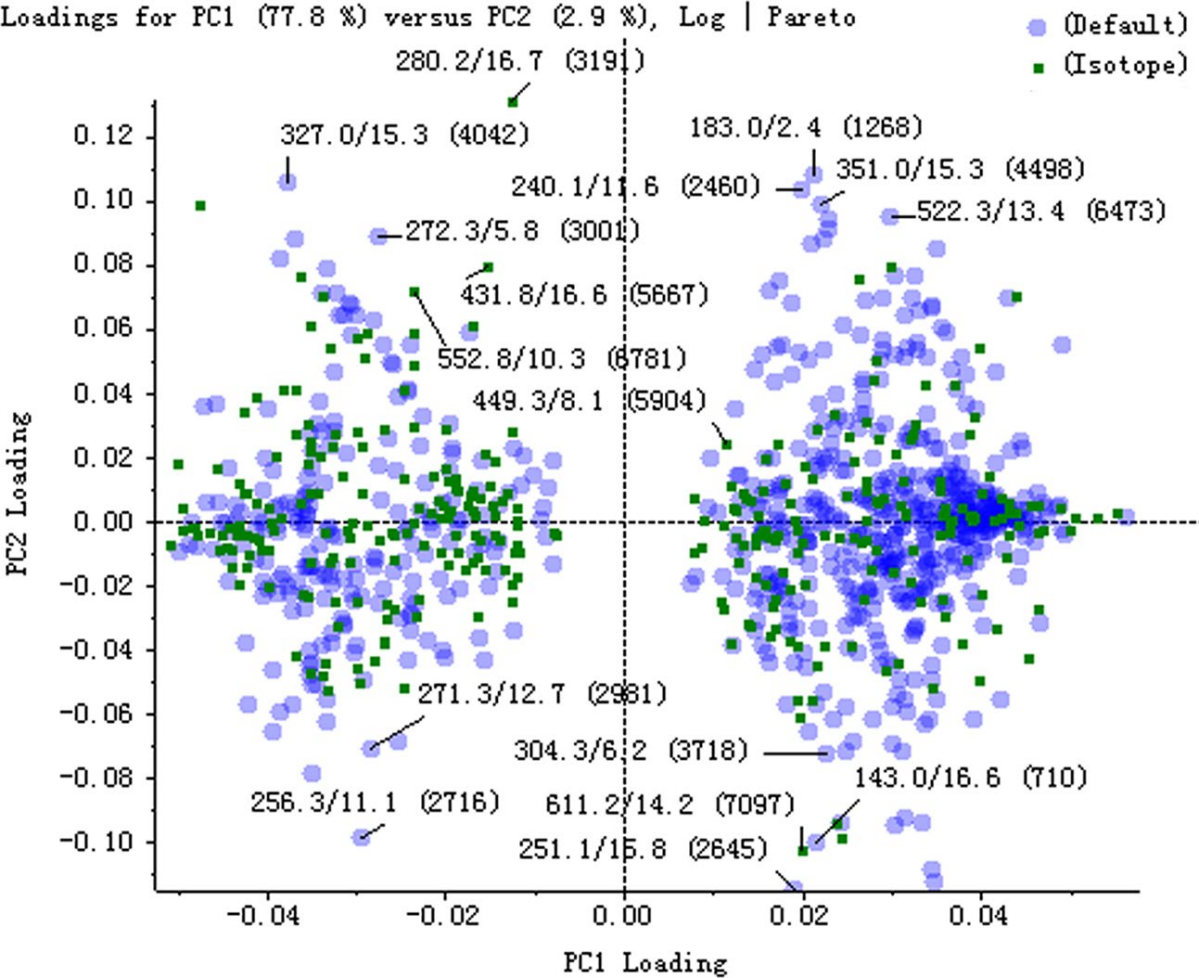
The loading plot of principal component analysis (PCA) between normal and model groups in positive ion mode.

The serum samples of rats in normal and model groups were monitored and supervised by the MarkerView^™^ software of AB SCIEX. The representative TICs of the serum samples from the normal group and model group in positive ion mode were shown in **Supplementary Figure 7**. The scores of rats in each group were obtained by PCA (**Figure 1**). The separation of the serum samples from model and normal groups was quite obvious. The spots of the model group distributed far from those of normal group. This result showed that the metabolism of rats in the model group was significantly altered. Through the analysis of the loading graph, the meaningful molecular weights of the endogenous molecules were found for each group (**Figure 2**). The results indicated that the intravenous injection of adriamycin could lead to kidney damage in rats and resulted in nephrotic syndrome.

#### Identification of Endogenous Metabolites

The potential biomarkers that made sense to grouping were finally found based on PCA score diagrams and loading diagrams. By comparing the possible biomarkers with the literatures and public online databases, 17 biomarkers in rat serum were preliminarily identified as shown in **Table 1**.

**Table 1:** Results of potential biomarkers detected in rat serum.

No.	Extract mass	Formula	Adduct	Retention time	Mass error	Matching rate	Identification	Database ID	Trend
1	115.0225	C_4_H_6_N_2_O_2_	[M+H]^+^	16.5	5.3	100%	l-3-Cyanoalanine	HMDB60245	Up
2	146.0590	C_6_H_11_NO_3_	[M+H]^+^	3.9	0.8	95%	Allysine	HMDB01263	Down
3	147.0493	C_5_H_10_N_2_O_3_	[M+H]^+^	16.5	0.3	97%	Alanylglycine	HMDB06899	Up
4	152.0354	C_8_H_9_NO_2_	[M+H]^+^	16.5	4.6	99%	2-Phenylglycine	HMDB02210	Down
5	184.0259	C_4_H_9_NO_5_S	[M+H]^+^	16.6	7.5	100%	l-Homocysteic acid	HMDB02205	Down
6	185.9871	C_3_H_8_NO_6_P	[M+H]^+^	16.6	−2.2	100%	Phosphoserine	HMDB00272	Up
7	192.0642	C_7_H_13_NO_3_S	[M+H]^+^	2.5	3	95%	*N*-Acetyl-l-methionine	HMDB11745	Up
8	203.1056	C_8_H_14_N_2_O_4_	[M+H]^+^	6.7	2.5	98%	Serinyl-proline	HMDB29047	Up
9	207.0860	C_7_H_14_N_2_O_3_S	[M+H]^+^	4.1	−0.1	100%	Methionyl-glycine	HMDB28973	Down
10	208.0879	C_11_H_13_NO_3_	[M+H]^+^	4.1	6.7	100%	*N*-Acetyl-l-phenylalanine	HMDB00512	Up
11	214.0379	C_4_H_8_NO_7_P	[M+H]^+^	16.7	0.9	100%	l-Aspartyl-4-phosphate	HMDB12250	Down
12	480.3467	C_24_H_50_NO_6_P	[M+H]^+^	10.6	−0.1	95%	Lyso PC (P-16:0)	HMDB10407	Down
13	536.3723	C_27_H_54_NO_7_P	[M+H]^+^	10.2	1.3	100%	Lyso PE [0:0/22:1 (13Z)]	HMDB11491	Up
14	559.4400	C_35_H_58_O_5_	[M+H]^+^	8.4	0.7	100%	DG [14:1 (9Z)/18:4 (6Z, 9Z, 12Z, 15Z)/0:0]	HMDB07048	Up
15	608.4654	C_32_H_66_NO_7_P	[M+H]^+^	13.1	4.7	100%	Lyso PC (24:0)	HMDB10405	Up
16	810.6001	C_46_H_84_NO_8_P	[M+H]^+^	15.3	3.2	100%	PC [22:4 (7Z, 10Z, 13Z, 16Z)/16:0]	HMDB08626	Up
17	832.5862	C_48_H_82_NO_8_P	[M+H]^+^	15.4	1.7	100%	PC [22:6 (4Z, 7Z, 10Z, 13Z, 16Z, 19Z)/18:1 (9Z)]	HMDB08729	Up

According to the results listed in **Table 1**, the endogenous components including l-3-cyanoalanine, alanylglycine, phosphoserine, *N*-acetyl-l-methionine, serinyl-proline, *N*-acetyl-l-phenylalanine, Lyso PE [0:0/22:1 (13Z)], Lyso PE [(6Z, 9Z, 12Z, 15Z)/0:0], PC (24:0), PC [22:4 (7Z, 10Z, 13Z, 16Z)/16:0], and PC [22:6 (4Z, 7Z, 10Z, 13Z, 16Z, 19Z, 19Z, 19Z)/18:1 (9Z)] in the nephrotic syndrome model group presented an upward trend. Meanwhile, allysine, 2-phenylglycine, l-homocysteic acid, methionyl-glycine, l-aspartyl-4-phosphate, and Lyso PC (P-16:0) in the nephrotic syndrome model group showed a downward trend. By comparing the changes of serum endogenous substances in the model group and the normal group, the significant increase or downregulation of these components was finally found. A relationship between endogenous components and nephrotic syndrome was discovered, which indicated that these endogenous metabolites could be used as characteristic biomarkers of nephrotic syndrome.

#### Metabolic Pathway Analysis

**Figure 3:**
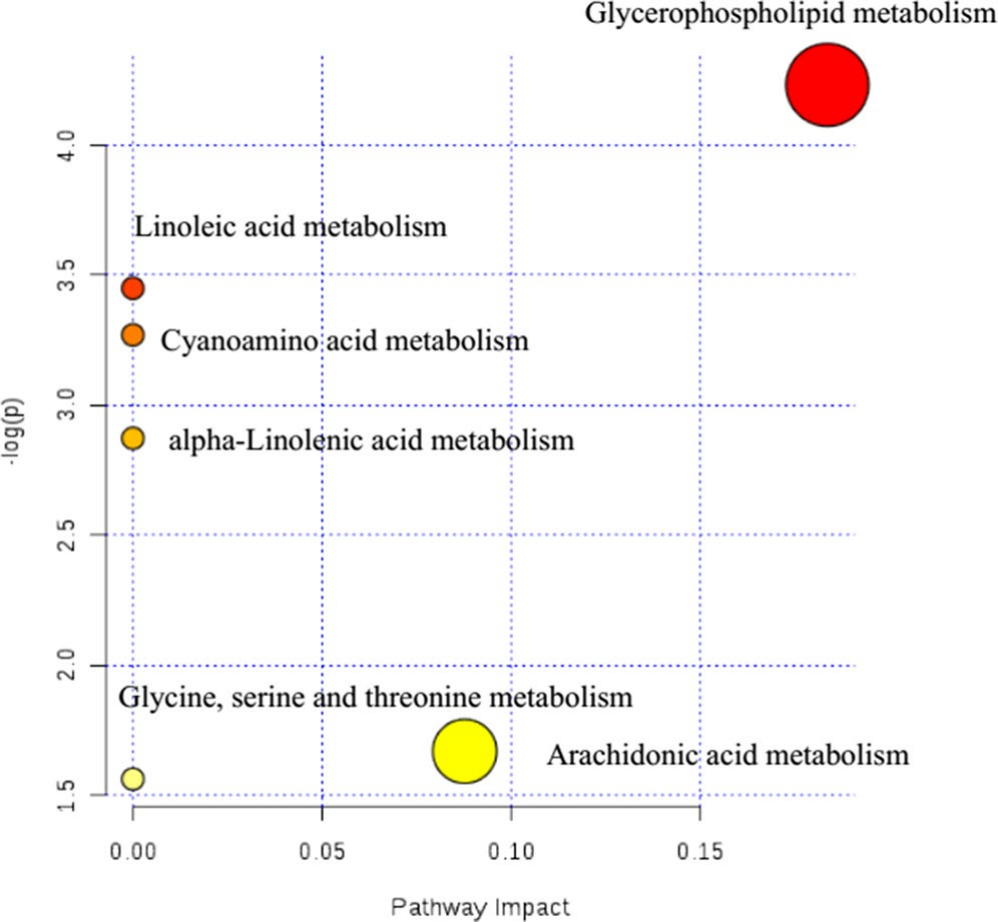
Summary of pathway analysis of serum samples collected from nephrotic syndrome rats.

Potential metabolic pathways of nephrotic syndrome were explored by imputing these 17 endogenous biomarkers listed in **Table 1** into MetaboAnalyst. Then, all the corresponding pathways were constructed according to *p* values given by pathway topology analysis (*x*-axis) (Xia and Wishart, 2011), with the most significant pathways colored in red (**Figure 3**).

In **Figure 3**, results showed that these 17 endogenous metabolites in rats involved in nephrotic syndrome were mainly related to six metabolic pathways. The six pathways included linoleic acid metabolism with an impact value of 0; cyanoamino acid metabolism with an impact value of 0; alpha-linolenic acid metabolism with an impact value of 0; glycine, serine, and threonine metabolism with an impact value of 0.087; arachidonic acid metabolism with an impact value of 0 and glycerophospholipid metabolism with an impact value of 0.183. It is suggested that the pathway of glycerophospholipid metabolism was the most important metabolic pathway for nephrotic syndrome formation, for the reason that the threshold of the impact value over 0.10 was commonly accepted as the most important metabolic pathway (Hoffmann et al., 2014). According to historical reports, lipid mediators, including eicosanoids, platelet-activating factor (PAF), and other chemotactic factors, have been proposed to contribute to leukocyte infiltration, mesangial proliferation, extracellular matrix protein production, vasoreactivity, and coagulation (Kees-Folts and Diamond, 1993). Further evidence of a relationship between PAF and the development of nephrotic syndrome was proven to be the marked elevation in plasma PAF-AH activity (Mimura et al., 1996).

#### Intensity-Time Variation of Biomarkers

**Figure 4:**
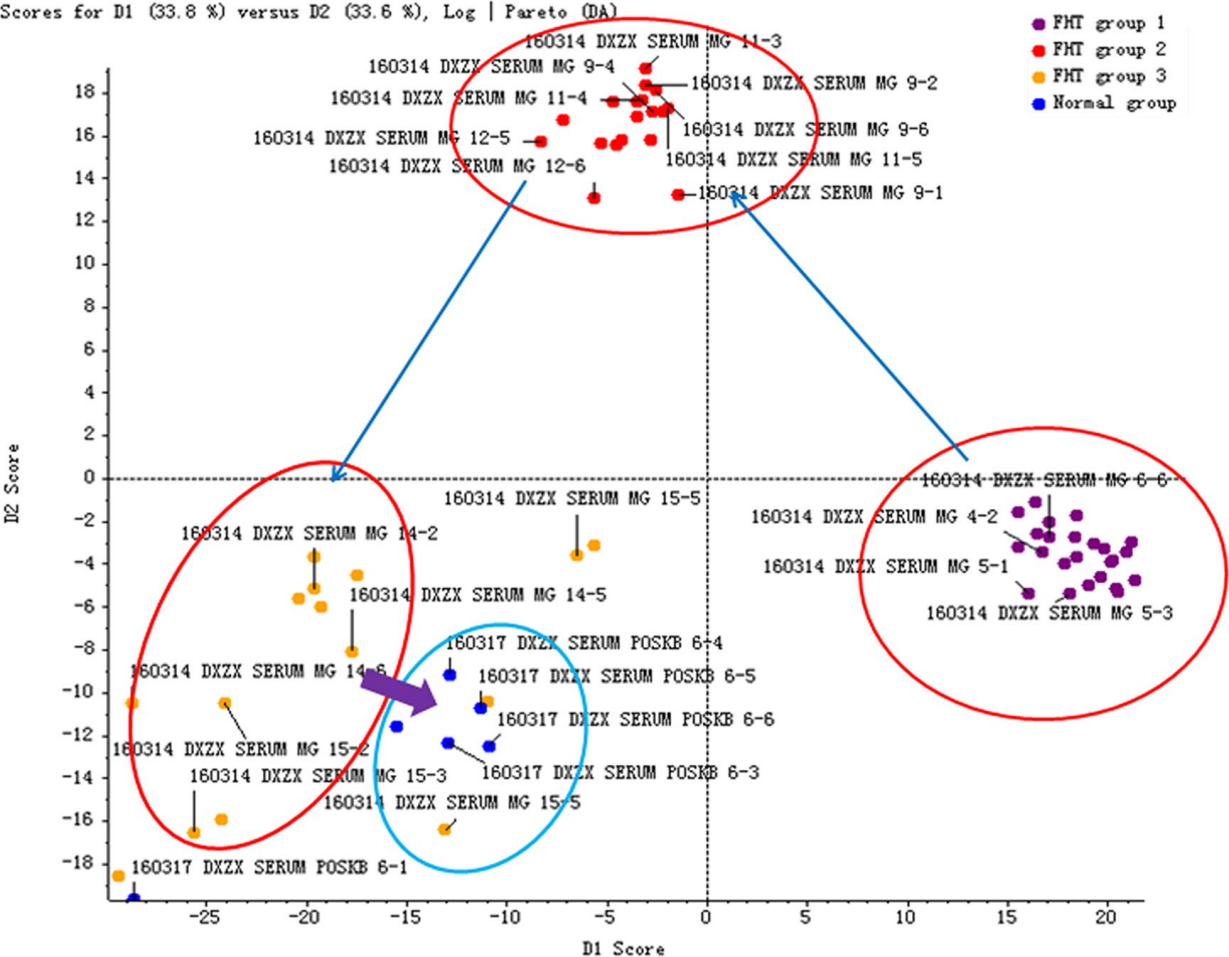
Time-related trajectory of metabolite patterns of nephrotic syndrome rats after FHT administration (FHT group 1 stands for the serum samples collected in an early drug medication: 23rd day; FHT group 2 stands for the serum samples collected in midterm drug medication: 44th day; FHT group 3 stands for the serum samples collected in later period of drug medication: 57th day).

In order to investigate the influence of FHT on the characteristic endogenous components, the rats with nephrotic syndrome were administrated with FHT once a day for 5 weeks. The blood samples of rats were collected at different time points. The intensity data of 17 endogenous biomarkers in the rat serum samples were extracted and analyzed by the PeakView^™^ software of AB SCIEX. The PCA score plot derived from the UHPLC-MS profiles of rat serum samples after FHT treatment in positive mode was shown in **Figure 4**. The plot shows a time-related trajectory of metabolite patterns at different time points after FHT treatment. And it can be seen that the metabolite pattern of rats was brought back to normal or near normal.

**Figure 5:**
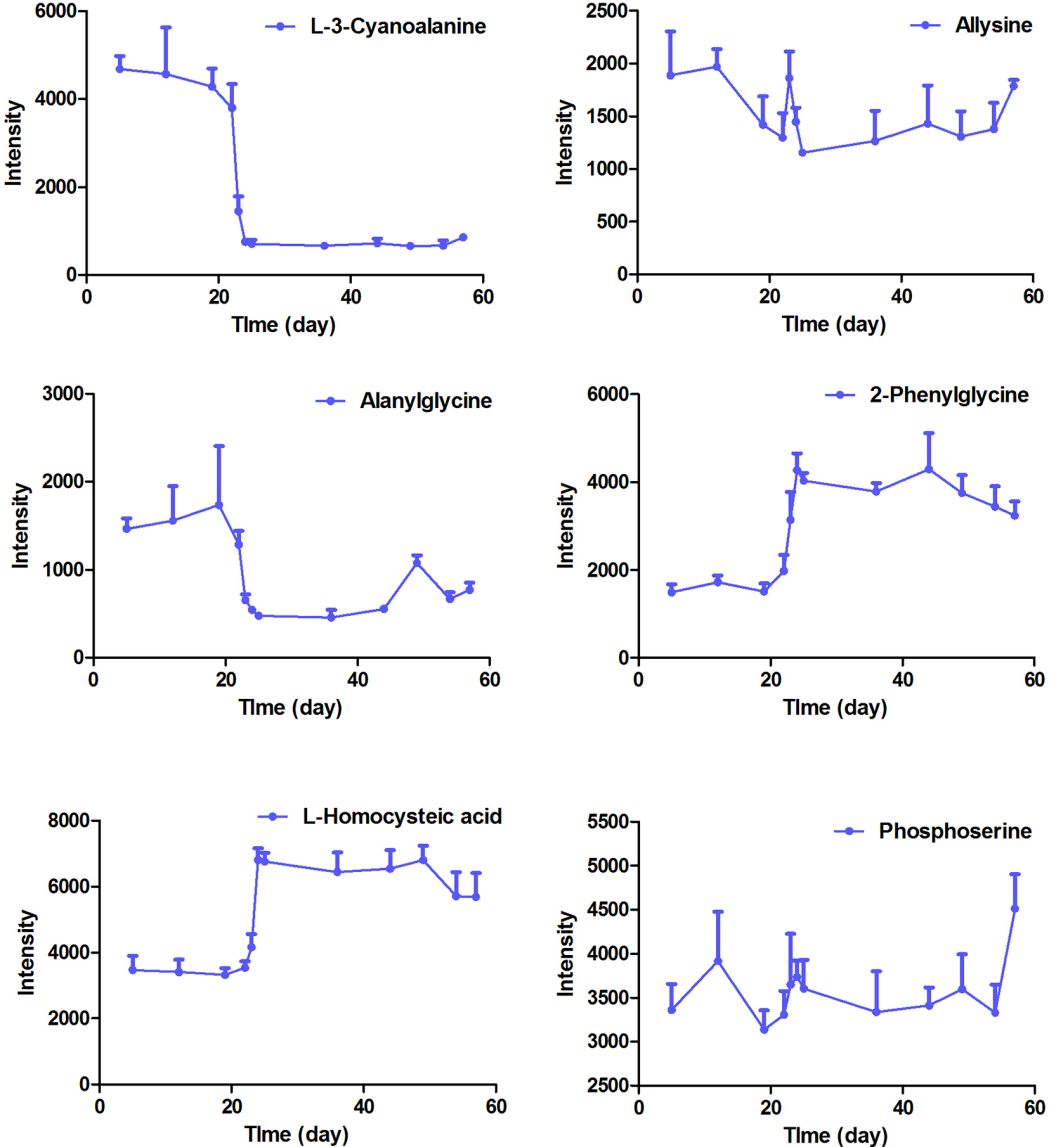
The intensity-time variation profiles of endogenous markers, including l-3-cyanoalanine, allysine, alanylglycine, 2-phenylglycine, l-homocysteic acid, and phosphoserine.

**Figure 6:**
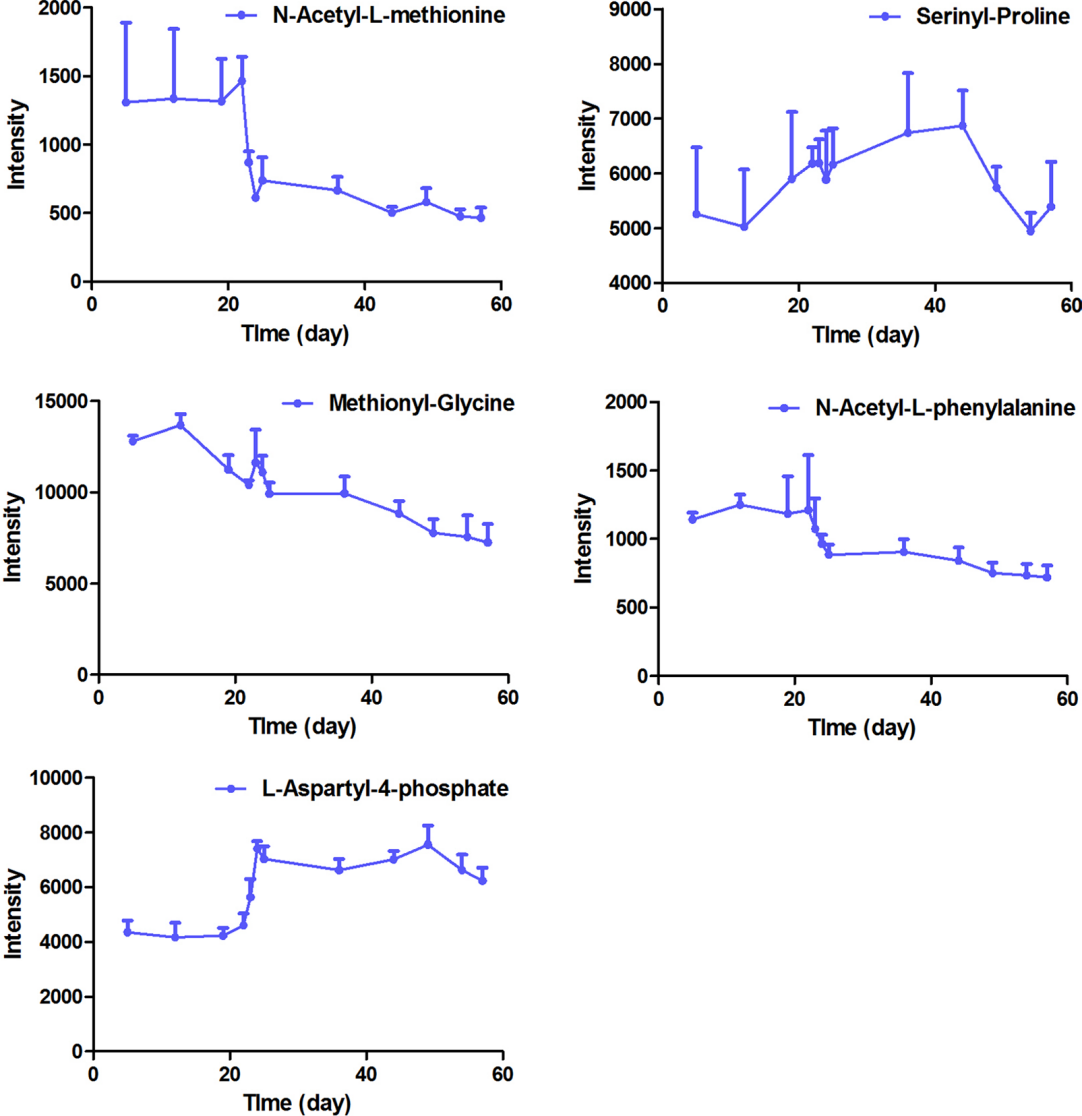
The intensity-time variation profiles of endogenous markers, including acetyl-l-methionine, serinyl-proline, methionyl-glycine, *N*-acetyl-l-phenylalanine, and l-aspartyl-4-phosphate.

**Figure 7:**
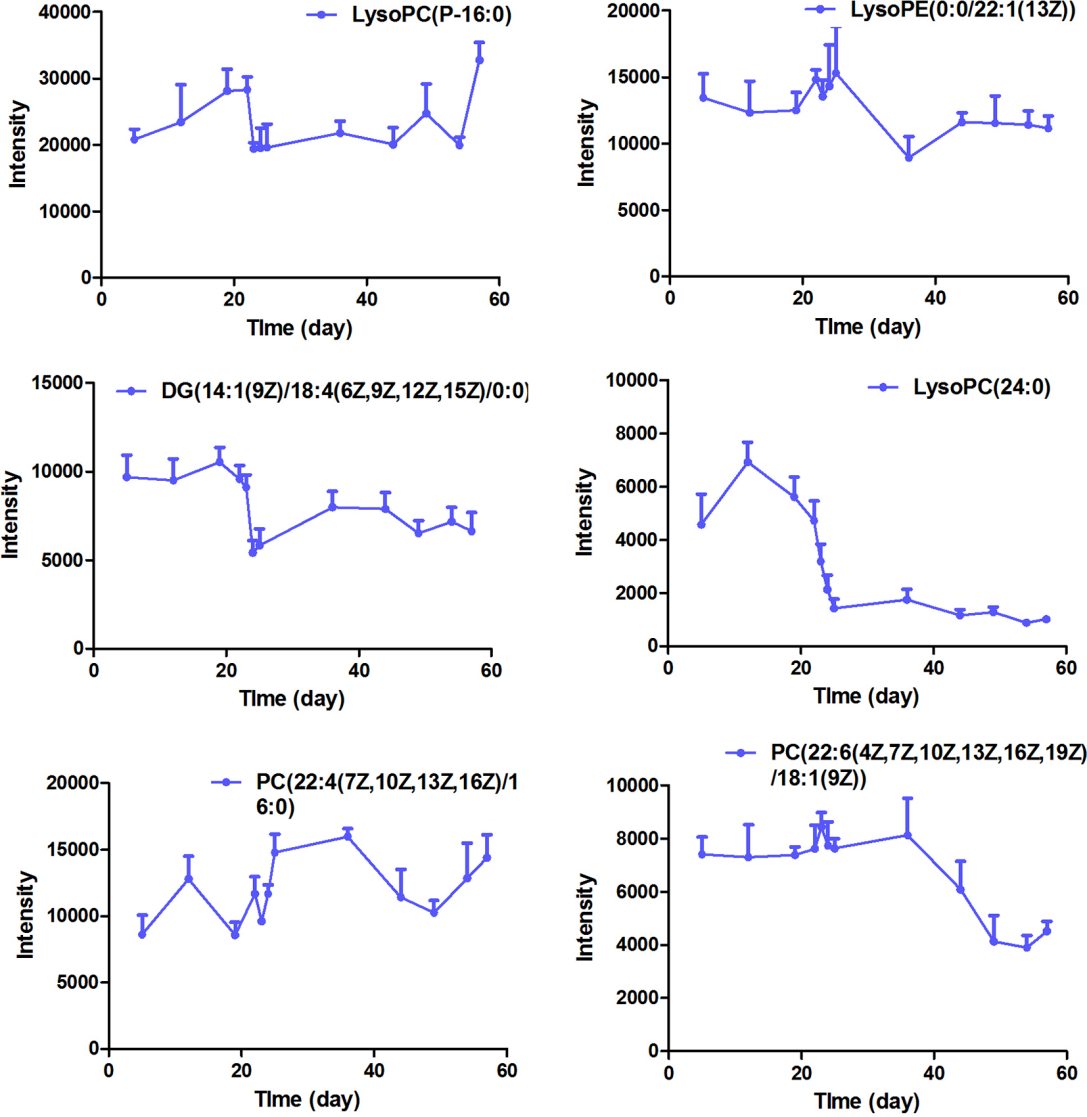
The intensity-time variation profiles of endogenous markers, including Lyso PC (P-16:0), Lyso PE [0:0/22:1 (13Z)], DG [14:1 (9Z)/18:4 (6Z, 9Z, 12Z, 15Z)/0:0], Lyso PC (24:0), PC [22:4 (7Z, 10Z, 13Z, 16Z)/16:0], and PC [22:6 (4Z, 7Z, 10Z, 13Z, 16Z, 19Z)/18:1 (9Z)].

The intensity-time variations of endogenous biomarkers were shown in **Figures 5**–**7**. The endogenous biomarkers containing 2-phenylglycine, l-homocysteic acid, and l-aspartyl-4-phosphate in FHT group showed a tendency to increase, while others like l-3-cyanoalanine, alanylglycine, *N*-acetyl-l-methionine, methionyl-glycine, *N*-acetyl-l-phenylalanine, Lyso PE [0:0/22:1 (13Z)], DG [14:1 (9Z)/18:4 (6Z, 9Z, 12Z, 15Z)/0:0], Lyso PC (24:0), and PC [22:6 (4Z, 7Z, 10Z, 13Z, 16Z, 19Z)/18:1 (9Z)] in the test group showed a tendency to decrease. Lipid metabolism disorder is usually related to the occurrence and development of nephropathy. Lyso PE [0:0/22:1 (13Z)], DG [14:1 (9Z)/18:4 (6Z, 9Z, 12Z, 15Z)/0:0], Lyso PC (24:0), and PC [22:6 (4Z, 7Z, 10Z, 13Z, 16Z, 19Z, 19Z)/18:1 (9Z)] all belonged to low-density lipoprotein, which plays an important role in lipid metabolism (Gaits et al., 1997). In model and test groups, the intensity of low-density lipoprotein showed a certain trend of change, which was consistent with the literature report (Gaits et al., 1997). The results indicated that FHT could regulate these endogenous substances to a certain degree.

### Dynamic Detection of FHT Relative Components

The FHT relative components in rat serum were identified by using the UHPLC-ESI-Q-TOF-MS technique based on high-resolution mass spectrometry of AB SCIEX Triple TOF^™^ 5600 and mass spectrum analysis software of PeakView^™^ based on the analysis of the chemical components in FHT developed at a previous stage; meanwhile, the metabolic pathway database in serum was established (Wang et al., 2015). In order to guarantee the accuracy of experimental results, the components in serum samples with an intensity less than 100 at various time points were dismissed. As a result, 43 components were selected to be involved in chemical spectrum–metabolic spectrum correlation analysis. The list of constituents in rat serum after oral administration of FHT by using the UHPLC-ESI-Q-TOF-MS method is shown in **Supplementary Table 2**. The typical ion chromatography spectrums of rat serum after being administered with FHT in positive and negative ion modes are shown in **Supplementary Figure 8**.

### Correlation Discovery Between Chemical and Metabolic Spectrums

**Figure 8:**
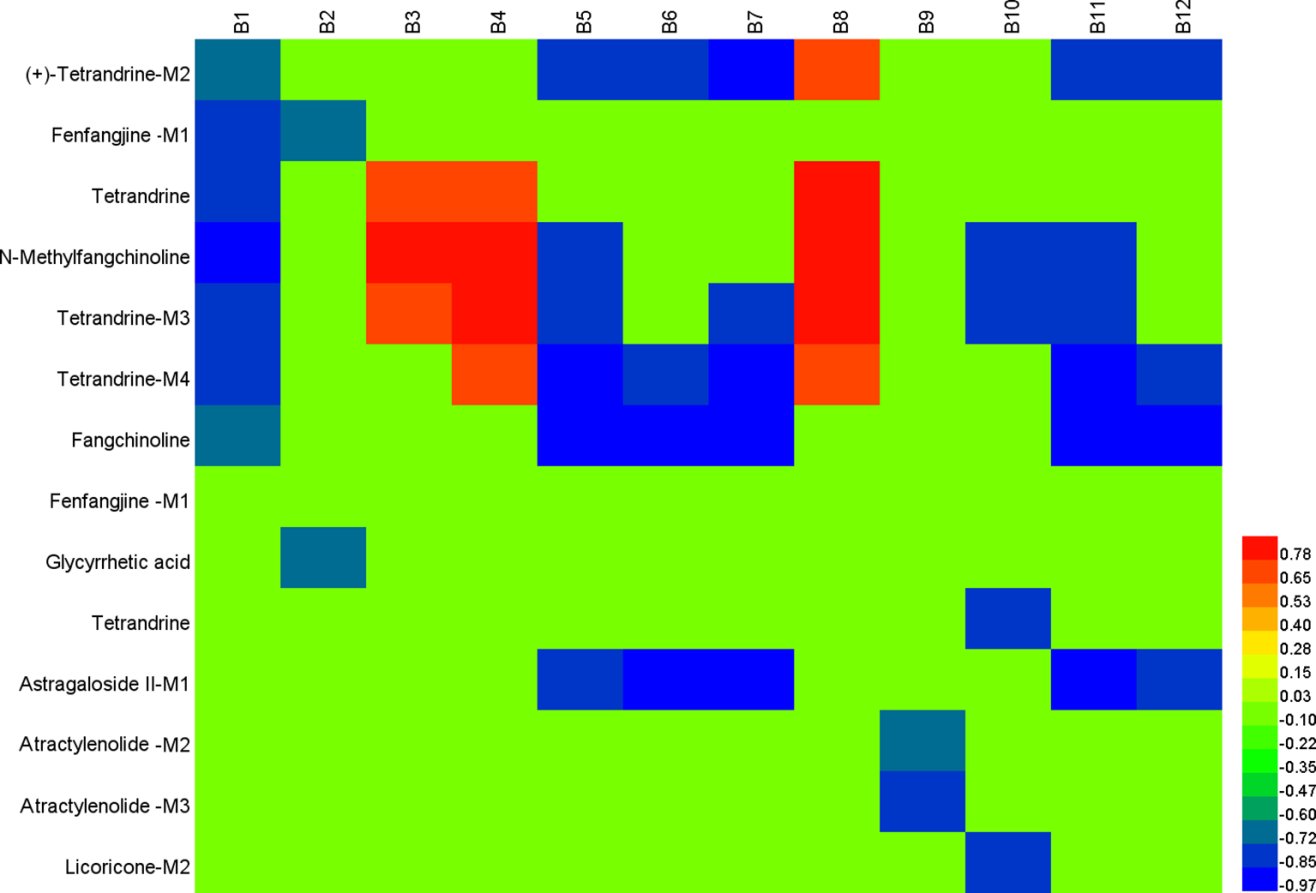
Correlation analysis map between potential biomarkers and Fangji Huangqi Tang (FHT) relative chemical components based on Pearson correlation coefficient using a color scale from significantly negative correlation (blue) to significantly positive correlation (red). B1, l-3-cyanoalanine; B2, alanylglycine; B3, 2-phenylglycine; B4, l-homocysteic acid; B5, *N*-acetyl-l-methionine; B6, methionyl-glycine; B7, *N*-acetyl-l-phenylalanine; B8, l-aspartyl-4-phosphate; B9, Lyso PE [0–0 draw/pay z (13)]; B10, DG [14:1 (z)/o (12 z, z, 6z, 9, 15 z)/0–0]; B11, Lyso PC (24:0); B12, PC [22:6 (4Z, 7Z, 10Z, 13Z, 16Z, 19Z, 19Z)/18:1 (9Z)].

The typical correlation analysis method was adopted to find out the relationships between chemical spectrum and metabolic spectrum with the purpose of screening the potential active components in FHT. The relationships of 43 relative compounds found in FHT and endogenous components, including l-3-cyanoalanine, alanylglycine, 2-phenylglycine, l-homocysteic acid, *N*-acetyl-l-methionine, methionyl-glycine, *N*-acetyl-l-phenylalanine, l-aspartyl-4-phosphate, Lyso PE [0–0 draw/pay z (13)], DG [14:1 (z)/o (12z, z, 6z, 9, 15 z)/0–0], Lyso PC (24:0), and PC [22:6 (4Z, 7Z, 10Z, 13Z, 16Z, 19Z, 19Z)/18:1 (9Z)], under a series of time points were investigated based on a chemical–metabolic spectrum correlation analysis. The results are shown in **Figure 8** and **Supplementary Tables 3**–**8**, which suggested that there was a significant correlation between the intensity changes of alkaloid components and the response intensity of differential endogenous substances in rat serum after drug administration, such as (+)-tetrandrine demethylation, fenfangjine G hydrogenation, tetrandrine, *N*-methylfangchinoline, tetrandrine demethylation, and fangchinoline. The alkaloids are the main active components of *S. tetrandra* S. Moore. This phenomenon was consistent with the traditional Chinese medical theory that “the medicine as sovereign is the core of the whole prescription.” At the same time, the study found that the alkaloids absorbed were transformed reciprocally through certain metabolic ways *in vivo*. It was speculated that these alkaloids and their metabolites worked efficiently by synergistic effect. In addition, the components of saponins were significantly correlated with the difference endogenous markers. The saponin compounds were derived from *A. membranaceus* (Fisch.) Bge—another medicine serving as sovereign in the whole prescription of FHT. Also, literatures already proved that saponins of *A. membranaceus* (Fisch.) Bge showed certain therapeutic effects on chronic renal disease (Yan et al., 2017; Zhou et al., 2017). Furthermore, the results of this work indicated that alkaloids and lactones in FHT could regulate lipid metabolism in rats. Lipid metabolism disorder was always believed to be an important pathological manifestation in the pathogenesis of nephrotic syndrome, because massive loss of proteinuria could result in lipid metabolism disorder. It was suggested that the alkaloids and lactones in FHT might treat nephrotic syndrome by the regulating lipid metabolism way in rats.

Active components in FHT for nephrotic syndrome treatment were screened out, including (+)-tetrandrine demethylation, fenfangjine G hydrogenation, tetrandrine, *N*-methylfangchinoline, tetrandrine demethylation, fangchinoline, glycyrrhetic acid, astragaloside II alcohol dehydration, atractylenolide III demethylation + hydrogenation, atractylenolide III demethylation + hydrogenation, and licoricone-*N*-acetylcysteine conjugation. From the results, we could see that they were prototypes as well as metabolites. Certainly, metabolites came from the corresponding prototypes. Tetrandrine, fenfangjine G, fangchinoline, glycyrrhetic acid, astragaloside II, atractylenolide III, and licoricone were finally found to be related to FHT efficacy on nephrotic syndrome treatment. Among them, tetrandrine, fenfangjine G, and fangchinoline came from *S. tetrandra* S. Moore (FJ); astragaloside II came from *A. membranaceus* Fisch. ex Bunge (HQ); atractylenolide III came from *A. macrocephala* Koidz. (BZ); and glycyrrhetic acid and licoricone came from *G. uralensis* Fisch. ex DC. (GC). According to our previous work, the quantities of these components in FHT were between 0.54 and 41.19 µg/ml (Wang et al., 2016), which are relatively high enough to be of sensible pharmacological relevance for herbal drugs. In all, chemical components in FHT, as well as their metabolites, were found to be the active materials for nephrotic syndrome treatment. These compounds were found to be involved in some metabolic pathways, including glycerophospholipid metabolism; linoleic acid metabolism; cyanoamino acid metabolism; alpha-linolenic acid metabolism; glycine, serine, and threonine metabolism; and arachidonic acid metabolism. For a long time, TCM was believed to exert pharmacological effects by multi-targets in multiforms. In this paper, prototypes of FHT relative components, as well as their metabolites, were found to be active substances for nephrotic syndrome treatment in rats by moderation effects in certain metabolic pathways, which proved supporting evidence for the speculation concerning the synergistic effect of the complex components in TCM.

## Conclusion

In this paper, a synthetic strategy combining the UHPLC-ESI-Q-TOF-MS chemical profiling technique and the CCA statistical method was successfully applied to screen out the potential active components in FHT for nephrotic syndrome treatment. Different from classical spectrum–effect relationship studies, the work in this paper concentrated on the relationships between drug relative chemical spectrums and serum biomarker profiles. It provided a meaningful reference mode to reveal the chemical material basis of complex TCM. The limitation of this work was that the specific ways for these components to exert their activities were not clearly explained. Further studies should be concentrated on the evaluation of the efficacy of these active components in FHT on the special pathways as well as their interactions.

## Data Availability Statement

The raw data supporting the conclusions of this manuscript will be made available by the authors, without undue reservation, to any qualified researcher.

## Ethics Statement

All the animal experiments were carried out according to the Guidelines for the Care and Use of Laboratory Animals, and were approved by the Animal Ethics Committee of Nanjing University of Chinese Medicine [Approval number: ACU-13(20151120)].

## Author Contributions

XL, X-CZ, and LX carried out the studies, performed the statistical analysis, and wrote the manuscript. Q-GZ participated in the animal experiments. XL and B-CC designed the work and revised the manuscript. All the authors have read and approved the final version.

## Funding

This work was financially supported by the National Natural Science Foundation of China (no. 81503207), Major Projects of Natural Science Foundation of Jiangsu Higher Education Institutions (no. 17KJA360006) and Jiangsu Province “333” project.

## Conflict of Interest

The authors declare that the research was conducted in the absence of any commercial or financial relationships that could be construed as a potential conflict of interest.
