# Supplementary figures and images for: Screening for Potential Active Components of Fangji Huangqi Tang on the Treatment of Nephrotic Syndrome by Using Integrated Metabolomics Based on “Correlations Between Chemical and Metabolic Profiles”
Source: Front Pharmacol. 2019 Oct 22;10:1261. doi: 10.3389/fphar.2019.01261 (PMC6817620; doi:10.3389/fphar.2019.01261)

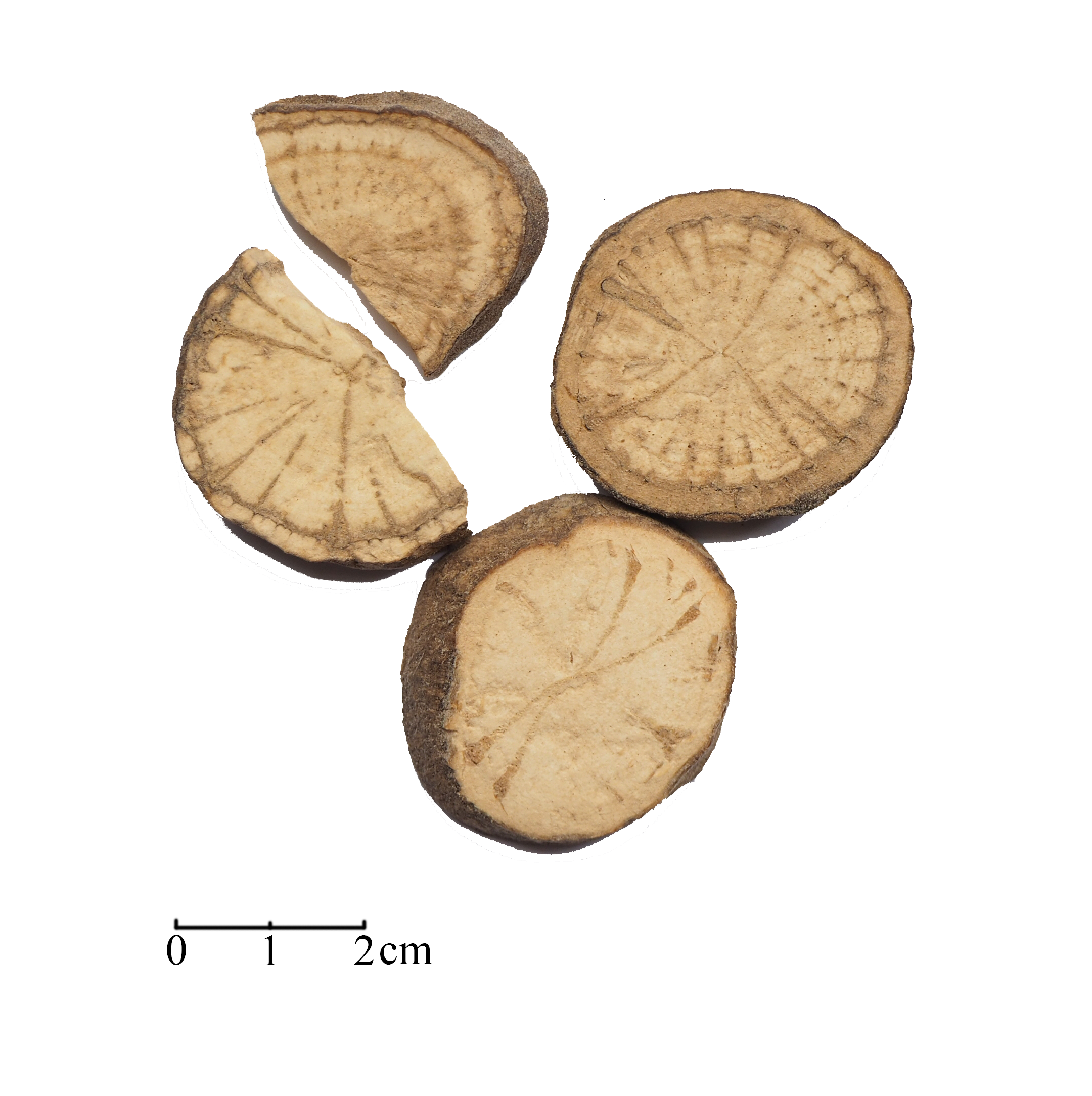

Supplement: Supplementary file 1 [file Image_1.tif]

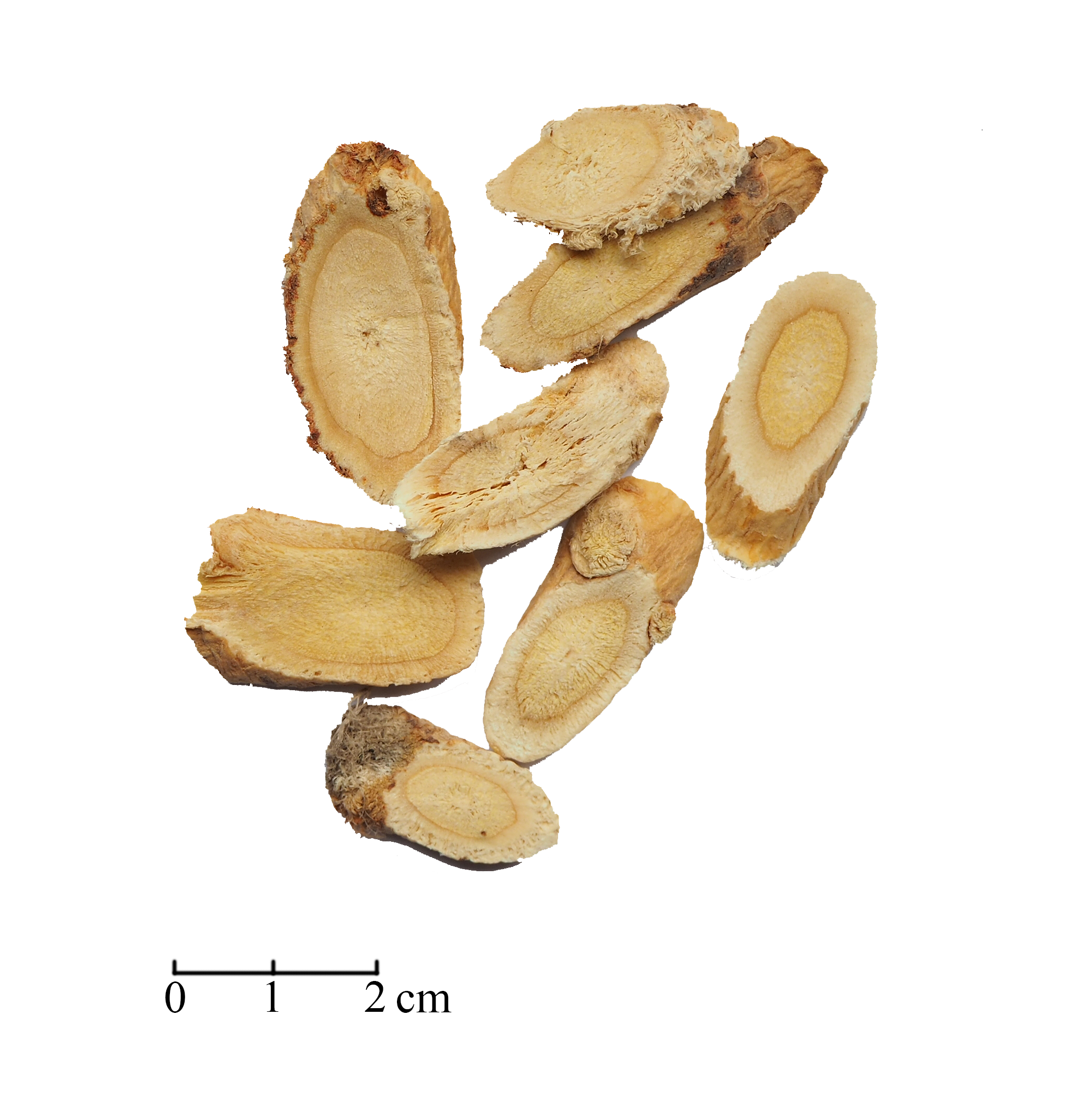

Supplement: Supplementary file 2 [file Image_2.tif]

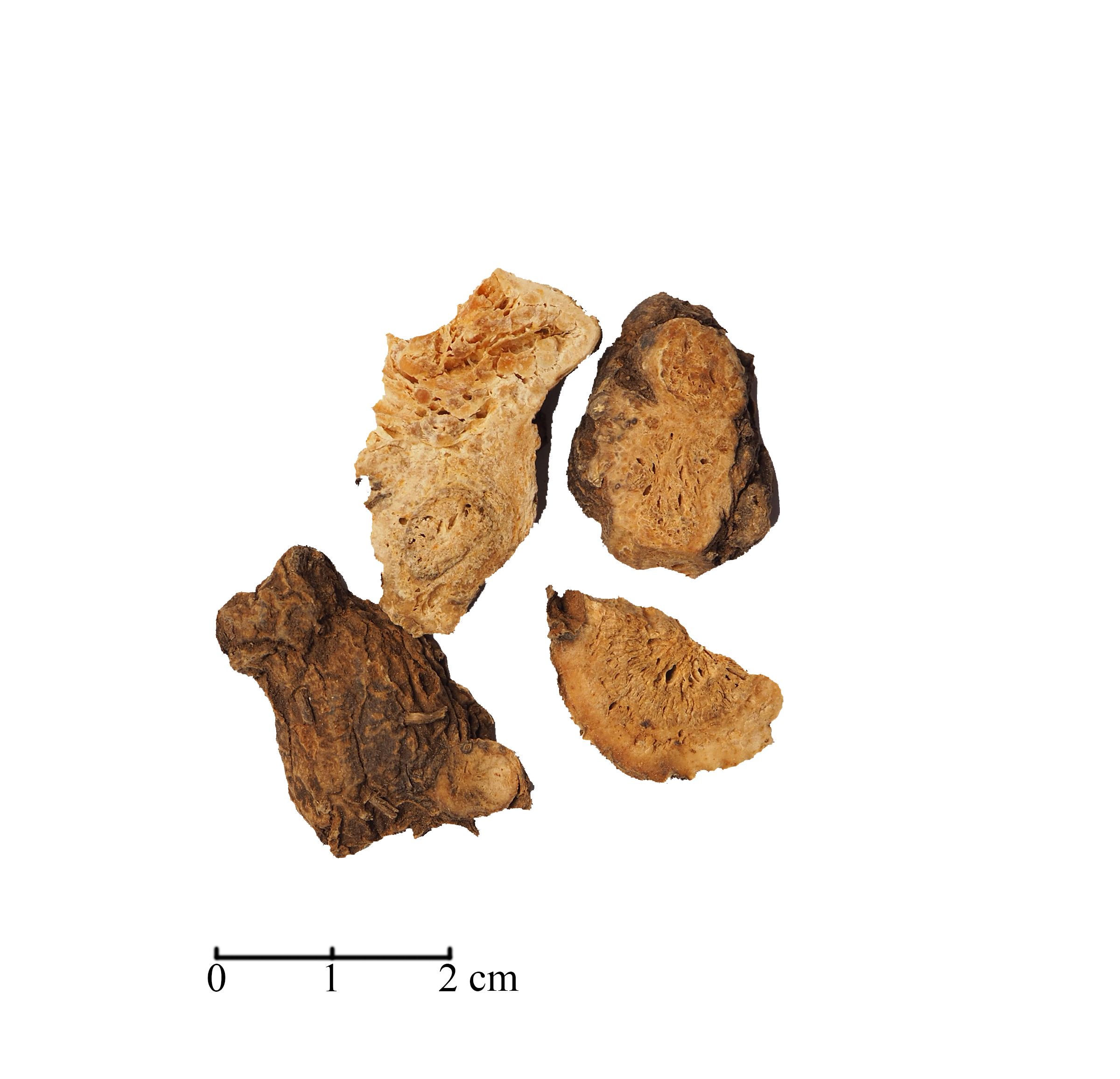

Supplement: Supplementary file 3 [file Image_3.tif]

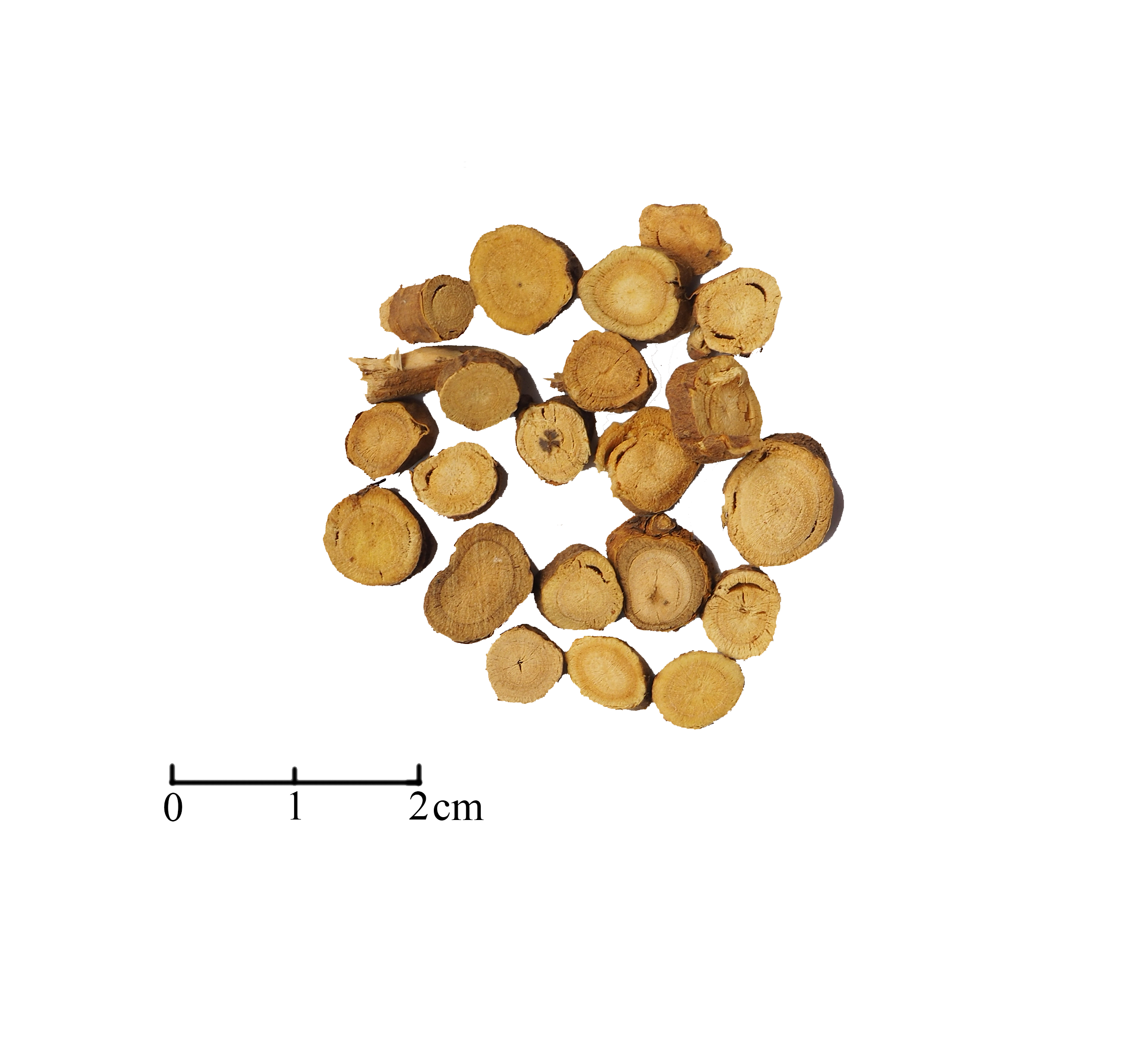

Supplement: Supplementary file 4 [file Image_4.tif]

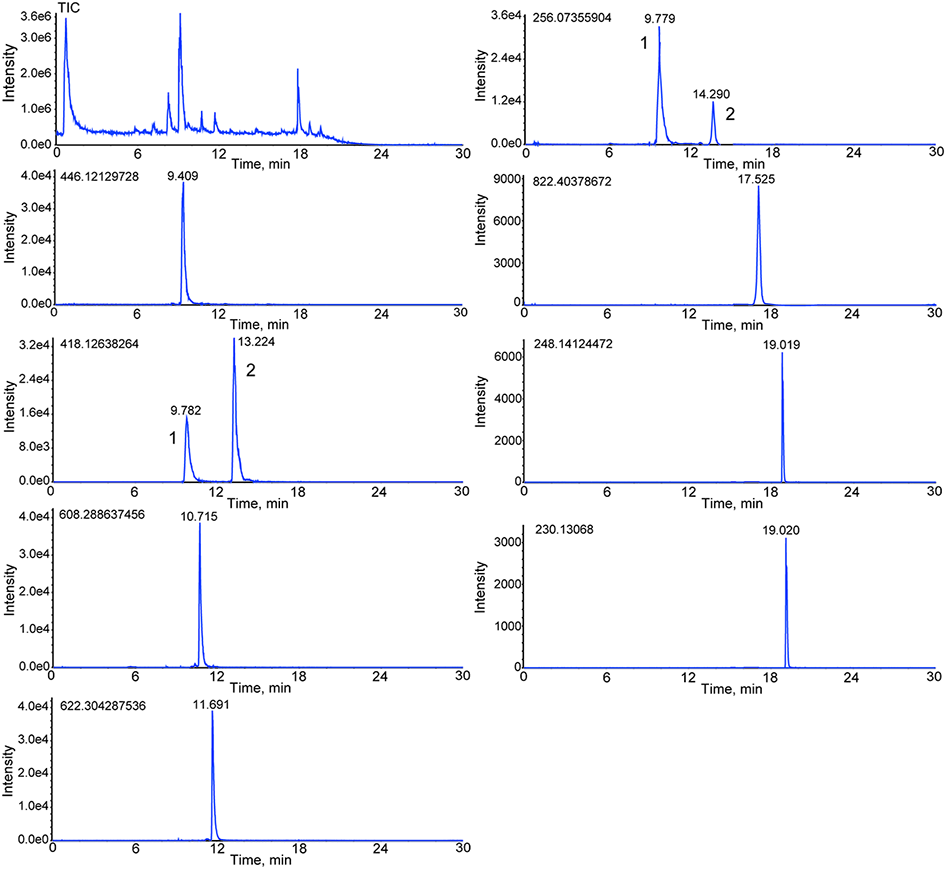

Supplement: Supplementary file 5 [file Image_5.tif]

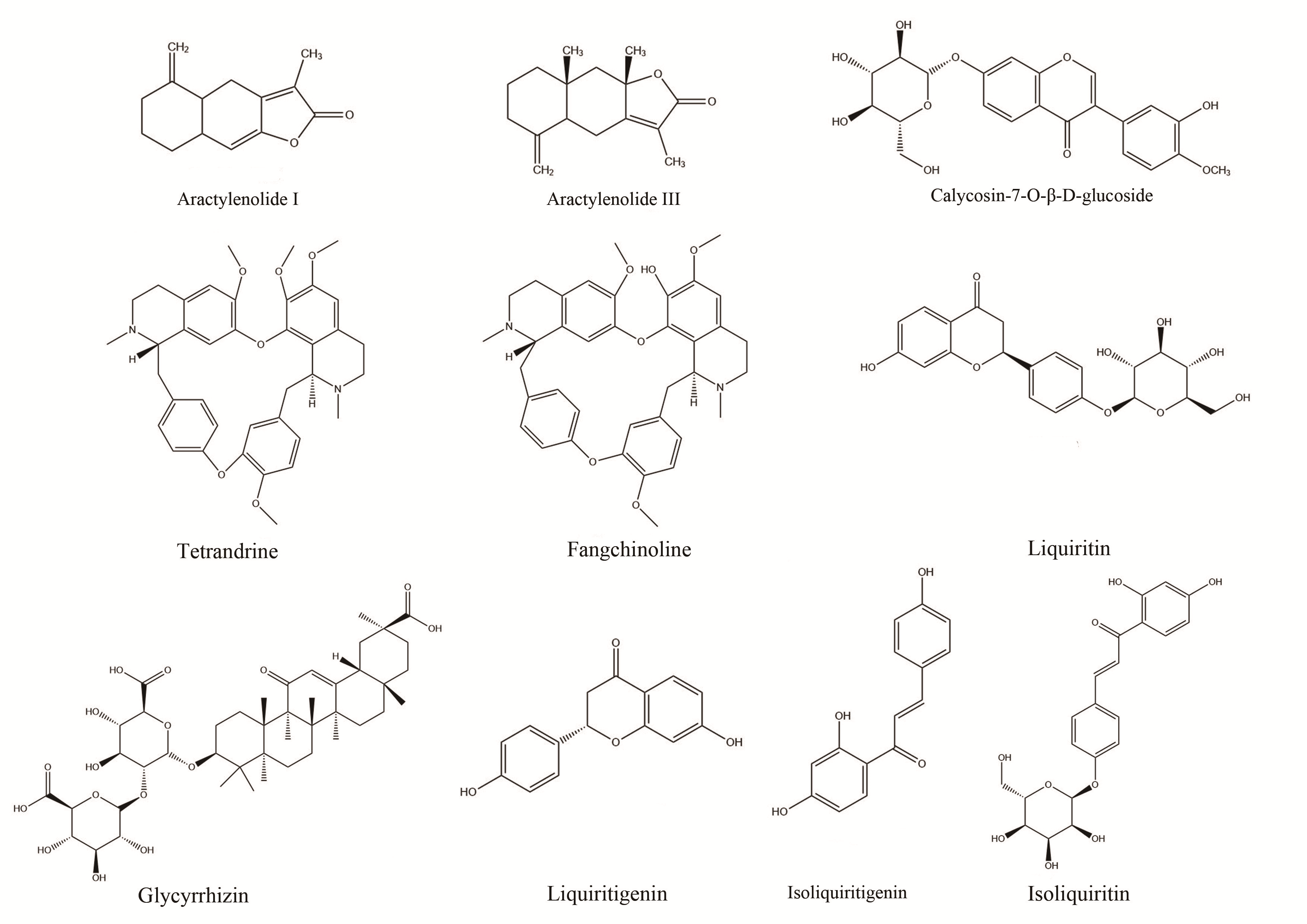

Supplement: Supplementary file 6 [file Image_6.tif]

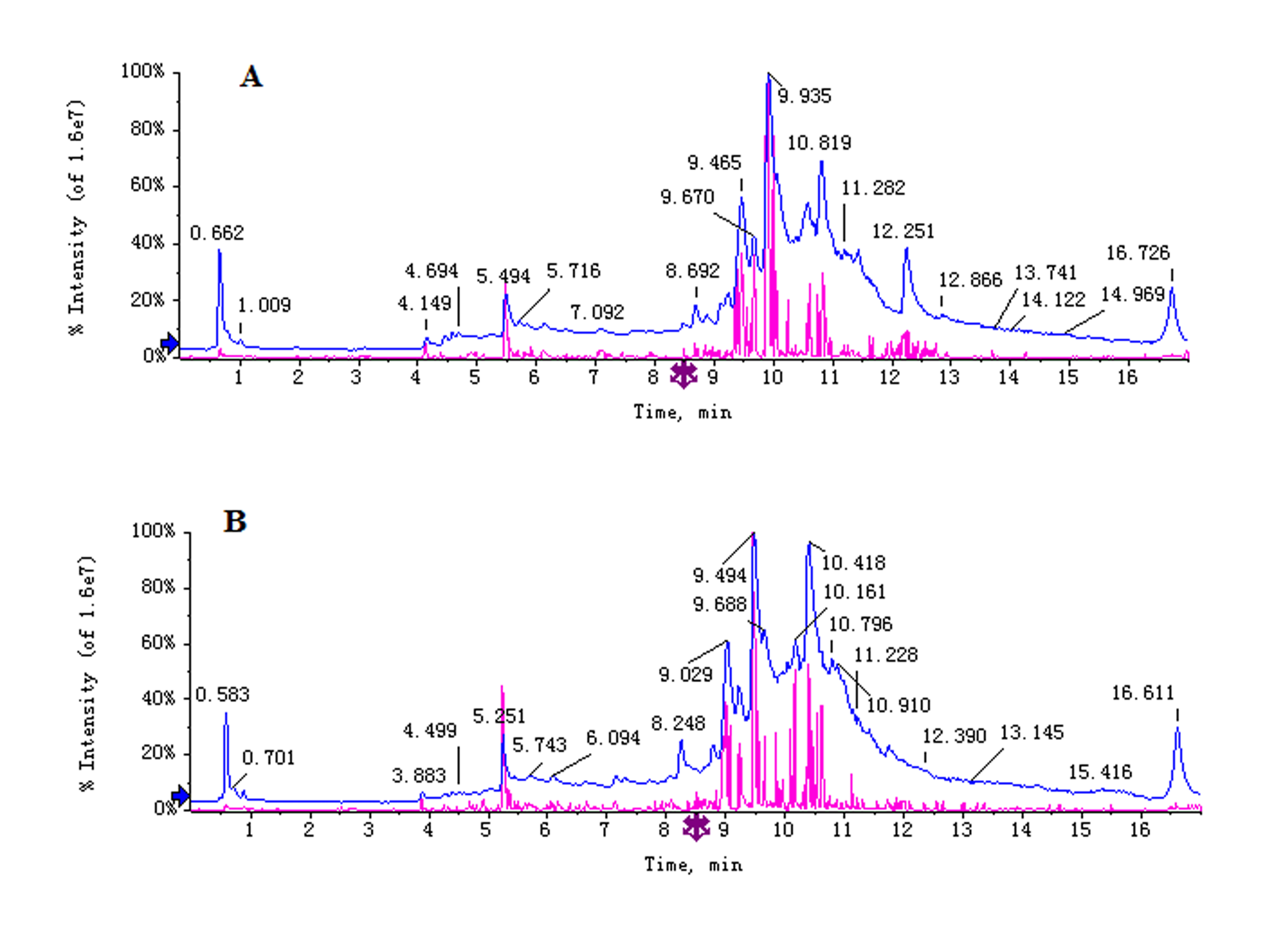

Supplement: Supplementary file 7 [file Image_7.tif]

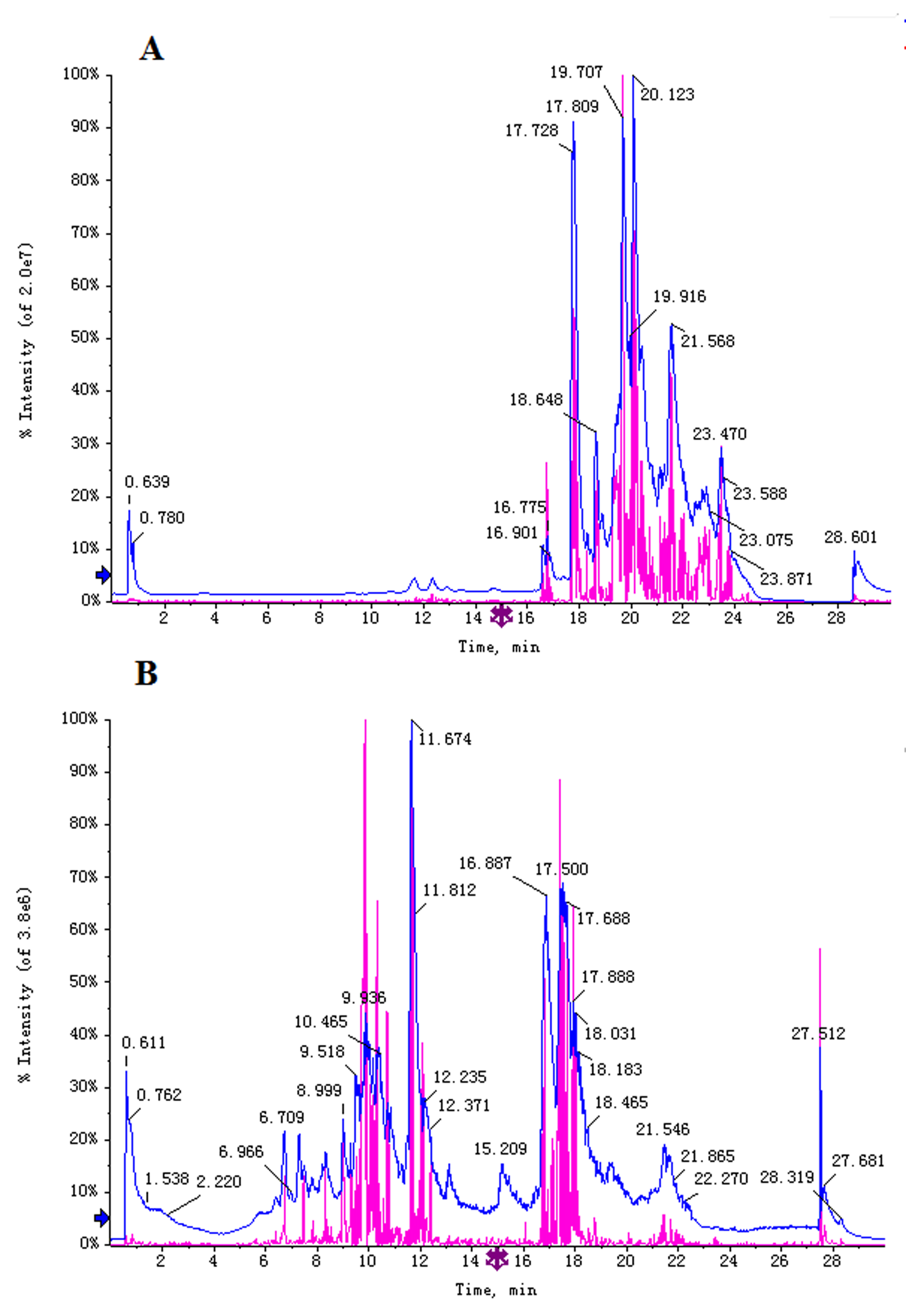

Supplement: Supplementary file 8 [file Image_8.tif]
