# Supplementary material for: Screening for Potential Active Components of Fangji Huangqi Tang on the Treatment of Nephrotic Syndrome by Using Integrated Metabolomics Based on “Correlations Between Chemical and Metabolic Profiles”
Source: Front Pharmacol. 2019 Oct 22;10:1261. doi: 10.3389/fphar.2019.01261 (PMC6817620; doi:10.3389/fphar.2019.01261)
Supplement: Supplementary file 9 [file Table_1.docx]

**Supplementary Table 1** Results of biomedical indexes assay in rats for nephrotic syndrome indication in nomal and model groups *(Mean ± SD, n=6)*.

| **Group** | **24 h urine protein (mg)** | **TC (mmol/L)** | **TG (mmol/L)** | **BUN (mmol/L)** | **Cys C (mmol/L)** |
| --- | --- | --- | --- | --- | --- |
| Normal | 34.99±2.28 | 1.75±0.34 | 0.78±0.04 | 5.45±0.41 | 65.08±5.33 |
| Model | 127.35±16.60*** | 2.61±0.52** | 1.59±0.80*** | 6.96±0.67*** | 185.91±7.34*** |

***^***^****P < 0.001 vs Normal group; 0.001 <****^**^****P < 0.005 vs Normal group.*

**Supplementary Table 2** The list of constituents in rat serum after oral administration of FHT by using UHPLC-ESI-Q-TOF-MS method.

| **No.** | **Name** | [**Metabolic way**](http://www.baidu.com/link?url=Bw3rKr1XQNAPfyKHd8sDz8ym3nDxYOKDa3HpcuZscwxTiRRNpXR4r5vrh31y7Q3EFZ9t-OWSE6eWkhPBaWfY-OnprH2GR9hT5yxB7nCTS3Io3aF5WojSTBvvZIgsrZMH) | **Formula** | **Source** |
| --- | --- | --- | --- | --- |
| 1 | Astragaloside I-M1 | Demethylation+desaturation | C_44_H_68_O_16_ | HQ |
| 2 | Astragaloside II-M1 | Alcohol dehydration | C_43_H_68_O_14_ | HQ |
| 3 | Tetrandrine-M2 | Demethylation | C_37_H_40_N_2_O_6_ | FJ |
| 4 | Licoricone-M2 | N-acetylcysteine conjugation | C_27_H_29_NO_9_S | GC |
| 5 | Methylnissolin 3-O-glucoside-M2 | Hydroxylation+desaturation | C_23_H_24_O_11_ | HQ |
| 6 | Formononetin-M1 | Hydroxylation+glucuronidation | C_22_H_20_O_11_ | HQ |
| 7 | Calycosin -7-O-D glucoside | Parent | C_22_H_22_O_10_ | HQ |
| 8 | Calycosin-M2 | Demethylation+glucuronidation | C_21_H_18_O_11_ | HQ |
| 9 | Calycosin -7-O-D glucoside-M5 | Hydroxylation+dehydration | C_22_H_20_O_10_ | HQ |
| 10 | Calycosin -7-O-D glucoside-M7 | Demethylation | C_21_H_20_O_10_ | HQ |
| 11 | Glyasperins C-M1 | 3*hydroxylation+desaturation | C_21_H_22_O_8_ | GC |
| 12 | Glyasperins C-M2 | 2*hydrogenation | C_21_H_28_O_5_ | GC |
| 13 | Glyasperins C-M3 | 2*hydrogenation | C_21_H_28_O_5_ | GC |
| 14 | (+) Cassythicine-M1 | Hydroxylation | C_19_H_19_NO_5_ | FJ |
| 15 | Fenfangjine F-M1 | Hydrogenation | C_19_H_21_NO_3_ | FJ |
| 16 | Formononetin-M4 | Hydroxylation | C_16_H_12_O_5_ | HQ |
| 17 | AtractylenolideⅡ-M2 | Acetylation | C_17_H_22_O_3_ | HQ |
| 18 | Odoration-M1 | Decarboxylation | C_16_H_14_O_4_ | GC |
| 19 | Atractylenolide Ⅲ-M1 | Hydroxylation | C_15_H_20_O_4_ | HQ |
| 20 | AtractylenolideⅡ-M3 | Hydroxylation+methylation | C_16_H_22_O_3_ | HQ |
| 21 | Formononetin-M5 | Demethylation+hydrogenation | C_15_H_12_O_4_ | HQ |
| 22 | AtractylenolideⅠ-M6 | Hydroxylation | C_15_H_18_O_3_ | HQ |
| 23 | Atractylenolide Ⅲ-M2 | Demethylation+hydrogenation | C_14_H_20_O_3_ | HQ |
| 24 | Atractylenolide Ⅲ-M3 | Demethylation+hydrogenation | C_14_H_20_O_3_ | HQ |
| 25 | Atractylenolide Ⅲ-M4 | Hydroxylation+deacetylation | C_13_H_18_O_3_ | HQ |
| 26 | Tetrandrine | Parent | C_38_H_42_N_2_O_6_ | FJ |
| 27 | Corydine | Parent | C_20_H_23_NO_4_ | FJ |
| 28 | N-Methylfangchinoline | Parent | C_38_H_43_N_2_O_6_ | FJ |
| 29 | Tetrandrine-M3 | Demethylation | C_37_H_40_N_2_O_6_ | FJ |
| 30 | Tetrandrine-M4 | Demethylation | C_37_H_40_N_2_O_6_ | FJ |
| 31 | Fangchinoline | Parent | C_37_H_40_N_2_O_6_ | FJ |
| 32 | Isoatractylenolide Ⅱ | Parent | C_15_H_20_O_2_ | BZ |
| 33 | Isoatractylenolide Ⅱ | Parent | C_15_H_20_O_2_ | BZ |
| 34 | Isoatractylenolide Ⅱ | Parent | C_15_H_20_O_2_ | BZ |
| 35 | Atractylenolide III | Parent | C_15_H_20_O_3_ | BZ |
| 36 | Dictamnoside A | Parent | C_21_H_36_O_9_ | BZ |
| 37 | Isoliguiritigenin | Parent | C_15_H_12_O_4_ | GC |
| 38 | Liguiritin | Parent | C_21_H_22_O_9_ | GC |
| 39 | Liguiritigenin | Parent | C_15_H_12_O_4_ | GC |
| 40 | Isoliguiritigenin | Parent | C_15_H_12_O_4_ | GC |
| 41 | 7 - hydroxy - 2 - methyl isoflavone | Parent | C_16_H_12_O_3_ | GC |
| 42 | Gancaonins X | Parent | C_21_H_22_O_4_ | GC |
| 43 | Glycyrrhetic acid | Parent | C_30_H_46_O_4_ | GC |

HQ: *Astragalus membranaceus* Fisch. ex Bunge; FJ: *Stephania tetrandra* S. Moore; GC: *Glycyrrhiza uralensis* Fisch. ex DC; BZ: *Atractylodes macrocephala* Koidz.

**Supplementary Table 3** The correlation coefficients between the peaks of potential effective components and endogenous components of L-3-cyanoalanine and alanylglycine (*n* = 9).

| **Endogenous components** | **L-3-cyanoalanine** | | | | | | | **Alanylglycine** | |
| --- | --- | --- | --- | --- | --- | --- | --- | --- | --- |
| Serum components | (+)-Tetrandrine-M2 | Fenfangjine-M1 | Tetrandrine | N-methylfangchinoline | Tetrandrine-M3 | Tetrandrine-M4 | Fangchinoline | Fenfangjine -M1 | Glycyrrhetic acid |
| Pearson relationship | -.693^*^ | -.760^*^ | -.763^*^ | -.882^**^ | -.830^**^ | -.762^*^ | -.720^*^ | -.605 | -.640 |
| Significance（Two-tailed） | .038 | .017 | .017 | .002 | .006 | .017 | .029 | .084 | .063 |
| N | 9 | 9 | 9 | 9 | 9 | 9 | 9 | 9 | 9 |

**stands for a significant correlation; ** stands for a very significant correlation.*

**Supplementary Table 4** The correlation coefficients between the peaks of potential effective components and endogenous components of 2-phenylglycine and L-homocysteic acid (*n* = 9).

| **Endogenous components** | **2-phenylglycine** | | | **L-homocysteic acid** | | | |
| --- | --- | --- | --- | --- | --- | --- | --- |
| Serum components | Tetrandrine | N-methylfangchinoline | Tetrandrine-M3 | Tetrandrine | N-methylfangchinoline | Tetrandrine-M3 | Tetrandrine-M4 |
| Pearson relationship | .776^*^ | .809^**^ | .680^*^ | .756^*^ | .811^**^ | .812^**^ | .668^*^ |
| Significance（Two-tailed） | .014 | .008 | .044 | .018 | .008 | .008 | .049 |
| N | 9 | 9 | 9 | 9 | 9 | 9 | 9 |

**stands for a significant correlation; **stands for a very significant correlation.*

**Supplementary Table 5** The correlation coefficients between the peaks of potential effective components and endogenous components of N-acetyl-L-methionine and methionyl-glycine (*n* = 9).

| **Endogenous components** | **N-acetyl-L-methionine** | | | | | | **Methionyl-glycine** | | | |
| --- | --- | --- | --- | --- | --- | --- | --- | --- | --- | --- |
| Serum components | Astragaloside II-M1 | (+)-Tetrandrine-M2 | N-methylfangchinoline | Tetrandrine-M3 | Tetrandrine-M4 | Fangchinoline | Astragaloside II-M1 | (+)-Tetrandrine-M2 | Tetrandrine-M4 | Fangchinoline |
| Pearson relationship | -.820^**^ | -.817^**^ | -.785^*^ | -.790^*^ | -.871^**^ | -.857^**^ | -.884^**^ | -.824^**^ | -.840^**^ | -.877^**^ |
| Significanc（Two-tailed） | .007 | .007 | .012 | .011 | .002 | .003 | .002 | .006 | .005 | .002 |
| N | 9 | 9 | 9 | 9 | 9 | 9 | 9 | 9 | 9 | 9 |

**stands for a significant correlation; ** stands for a very significant correlation.*

**Supplementary Table 6** The correlation coefficients between the peaks of potential effective components and endogenous components of N-acetyl-L-phenylalanine and L-aspartyl-4-phosphate (*n* = 9).

| **Endogenous components** | **N-acetyl-L-phenylalanine** | | | | | **L-aspartyl-4-phosphate** | | | | |
| --- | --- | --- | --- | --- | --- | --- | --- | --- | --- | --- |
| Serum components | Astragaloside II-M1 | (+)-Tetrandrine-M2 | Tetrandrine-M3 | Tetrandrine-M4 | Fangchinoline | (+)-Tetrandrine-M2 | Tetrandrine | N-Methylfangchinoline | Tetrandrine-M3 | Tetrandrine-M4 |
| Pearson relationship | -.950^**^ | -.908^**^ | -.809^**^ | -.970^**^ | -.972^**^ | .675^*^ | .854^**^ | .905^**^ | .882^**^ | .714^*^ |
| Significance（Two-tailed） | .000 | .001 | .008 | .000 | .000 | .046 | .003 | .001 | .002 | .031 |
| N | 9 | 9 | 9 | 9 | 9 | 9 | 9 | 9 | 9 | 9 |

**stands for a significant correlation; **stands for a very significant correlation.*

**Supplementary Table 7** The correlation coefficients between the peaks of potential effective components and endogenous components of Lyso PE (0:0/22:1(13Z)) and DG (14:1(9Z)/18:4(6Z,9Z,12Z,15Z)/0:0) (*n* = 9).

| **Endogenous components** | **Lyso PE (0:0/22:1(13Z))** | | **DG (14:1(9Z)/18:4(6Z,9Z,12Z,15Z)/0:0)** | | | |
| --- | --- | --- | --- | --- | --- | --- |
| Serum components | Atractylenolide Ⅲ-M2 | Atractylenolide Ⅲ-M3 | Licoricone-M2 | Tetrandrine | N-methylfangchinoline | Tetrandrine-M3 |
| Pearson relationship | -.707^*^ | -.772^*^ | -.793^*^ | -.786^*^ | -.828^**^ | -.832^**^ |
| Significance（Two-tailed） | .033 | .015 | .011 | .012 | .006 | .005 |
| N | 9 | 9 | 9 | 9 | 9 | 9 |

**stands for a significant correlation; **stands for a very significant correlation.*

**Supplementary Table 8** The correlation coefficients between the peaks of potential effective components and endogenous components of Lyso PC (24:0) and PC (22:6(4Z,7Z,10Z,13Z,16Z,19Z)/18:1(9Z)) (*n* = 9).

| **Endogenous components** | **Lyso PC (24:0)** | | | | | | **PC (22:6(4Z,7Z,10Z,13Z,16Z,19Z)/18:1(9Z))** | | | |
| --- | --- | --- | --- | --- | --- | --- | --- | --- | --- | --- |
| Serum components | Astragaloside II-M1 | (+)-Tetrandrine-M2 | N-methylfangchinoline | Tetrandrine-M3 | Tetrandrine-M4 | Fangchinoline | Astragaloside II-M1 | (+)-Tetrandrine-M2 | Tetrandrine-M4 | Fangchinoline |
| Pearson relationship | -.856^**^ | -.847^**^ | -.755^*^ | -.814^**^ | -.904^**^ | -.894^**^ | -.850^**^ | -.838^**^ | -.845^**^ | -.875^**^ |
| Significance（Two-tailed） | .003 | .004 | .019 | .008 | .001 | .001 | .004 | .005 | .004 | .002 |
| N | 9 | 9 | 9 | 9 | 9 | 9 | 9 | 9 | 9 | 9 |

**stands for a significant correlation; ** stands for a very significant correlation.*
